# Humans correctly assign emotional valence of rat vocalizations

**DOI:** 10.3389/fpsyg.2026.1769385

**Published:** 2026-05-20

**Authors:** Eduard Maier, Shimpei Ishiyama, Valery Grinevich, Michael Brecht

**Affiliations:** 1 Department of Neuropeptide Research in Psychiatry, Central Institute for Mental Health, University of Heidelberg, Mannheim, Germany; 2 German Center for Mental Health (DZPG), Mannheim-Heidelberg-Ulm, Mannheim, Germany; 3 Bernstein Center for Computational Neuroscience Berlin, Humboldt-Universität zu Berlin, Berlin, Germany; 4 NeuroCure Cluster of Excellence, Humboldt-Universität zu Berlin, Berlin, Germany; 5 Einstein Center for Neurosciences Berlin, Berlin, Germany

**Keywords:** auditory perception, emotional valence, evolution, interspecies communication, vocalizations

## Abstract

Emotions are signalled among conspecifics but also to other species to facilitate ethologically meaningful responses towards external and internal cues. An unresolved question, however, is whether the universality of emotional recognition extends to vocalizations that lie outside a species’ natural auditory range. Here we asked whether humans perceive emotional content of frequency-shifted rat ultrasonic vocalizations. We found that human subjects correctly assigned emotional valence to the artificially transposed vocalizations, with certain dependencies on the degree of the frequency shift. This suggest that vocal emotional communication is highly conserved across species and that continuous sensory exposure is unlikely to be required for evolutionary preservation of these vocal expressions.

## Introduction

As Darwin already suggested over a century ago, many emotions, particularly those that are signalled through vocalizations, are inherited rather than learned, because they (i) can be triggered by appropriate stimuli even in new-borns, and (ii) are shared across species (Darwin, 1872). We know today that cross-species vocal content recognition is a widespread phenomenon (Lingle and Riede, 2014; Lehoczki et al., 2020; Maigrot et al., 2022; Greenall et al., 2022; Lefèvre et al., 2025), and therefore, it is not unexpected that humans can recognize the vocal emotional content signalled by other species (Greenall et al., 2022; Scheumann et al., 2017; Tallet et al., 2010; Farago et al., 2014; Filippi et al., 2017). No comparable studies exist on rodent laboratory animals, such as rats, despite evidence from behavioural and pharmacological studies that rat ultrasonic vocalizations (USVs) can be broadly categorized in 50 kHz- and 22 kHz USVs, respectively reflecting positive- and negative valence emotional states (Knutson et al., 2002; Wöhr and Schwarting, 2007; Burgdorf et al., 2008; Brudzynski, 2014; Bonauto et al., 2023; Panksepp and Burgdorf, 2003; Brudzynski, 2019).

50 kHz USVs are typically high frequency calls around 35–72 kHz (Brudzynski, 2005). They are relatively short (30–50 ms) and often show frequency modulation (Burgdorf et al., 2008). However, some are unmodulated and are subcategorized as ‘flat’ 50 kHz USVs, which seem to be emitted during rather neutral contexts (Burgdorf et al., 2008). Frequency-modulated 50 kHz USVs – as presented in this study – are emitted during appetitive encounters with another individual (Burgdorf et al., 2008) and induce approach behaviour (Wöhr and Schwarting, 2007; Burgdorf et al., 2008). In contrast, 22 kHz USVs carry spectral energy around 18–35 kHz (Brudzynski, 2005). These USVs are typically long (0.3–3.4 s) (Brudzynski, 2005) and have comparably little frequency modulation (Burgdorf et al., 2008). These USVs are typically emitted during fearful (Fendt et al., 2018) or aggressive encounters and serve as an alarm signal – they usually evoke an avoidance response (Wöhr and Schwarting, 2007; Burgdorf et al., 2008).

Pharmacological studies are in line with the above-mentioned observations; 50 kHz USVs are linked to dopaminergic transmission, which is implicated in reward-related brain functions. For example, amphetamine injections in the nucleus accumbens evokes 50 kHz USV emission and dopamine antagonists reduce them (Thompson et al., 2006). Furthermore, electrical stimulation of the mesolimbic pathway and other reward-related areas evokes 50 kHz USV emission (Burgdorf et al., 2007). In contrast, 22 kHz USVs are linked to central cholinergic transmission which has been linked to defensive- or anxiety-like states (Brudzynski, 2015). In line with this, stimulation of brain areas that are part of fear/defence circuits, such as the amygdala and the periaqueductal gray mainly lead to the generation of 22 kHz USVs (Kim et al., 2013).

Although the dichotomy between 50 kHz and 22 kHz USVs is often described in terms of positive versus negative valence, both call types show variability depending on social setting, arousal level, ambivalence of the stimulus and individual differences. For example, a subset of 50 kHz USVs – “combined USVs” – are frequently emitted during heterospecific play contexts and contain frequency components close to 22 kHz (Ishiyama and Brecht, 2016). Also, counterintuitively, 22 kHz USVs are emitted following ejaculation (Burgdorf et al., 2008; Barfield and Geyer, 1972).

Summarized, the literature reviewed above suggests that 50 kHz USVs are linked to positive emotional states, while 22 kHz USVs are linked to negative emotional states.

Given this association between USV type and emotional state, rat USVs may be suited for cross-species emotional recognition investigations involving humans. However, USVs cannot be perceived by humans in natural settings. To overcome this issue, we transposed rat USVs to the human audible hearing range, and asked human individuals to rate the emotional content of these vocalizations. As the physical characteristics of naturally occurring rat USVs as well as human auditory perception must have evolved independently from each other our experiment provides insights into the rarely asked question whether sensory exposure is required to conserve vocal emotional content across evolution.

## Materials and methods

All methods were carried out in accordance with relevant guidelines and regulations. Further, all experimental protocols were approved by the Ethics-Commission II – University of Heidelberg, Medical Faculty Mannheim (Permit No: 2024–677) and by the Regierungspräsidium Karlsruhe (Permit No: G55-23). Informed consent was obtained from all subjects. Subjects displayed various ages, had different native languages, gender and professional background (Supplementary Tables 1, 2).

**Figure 1:**
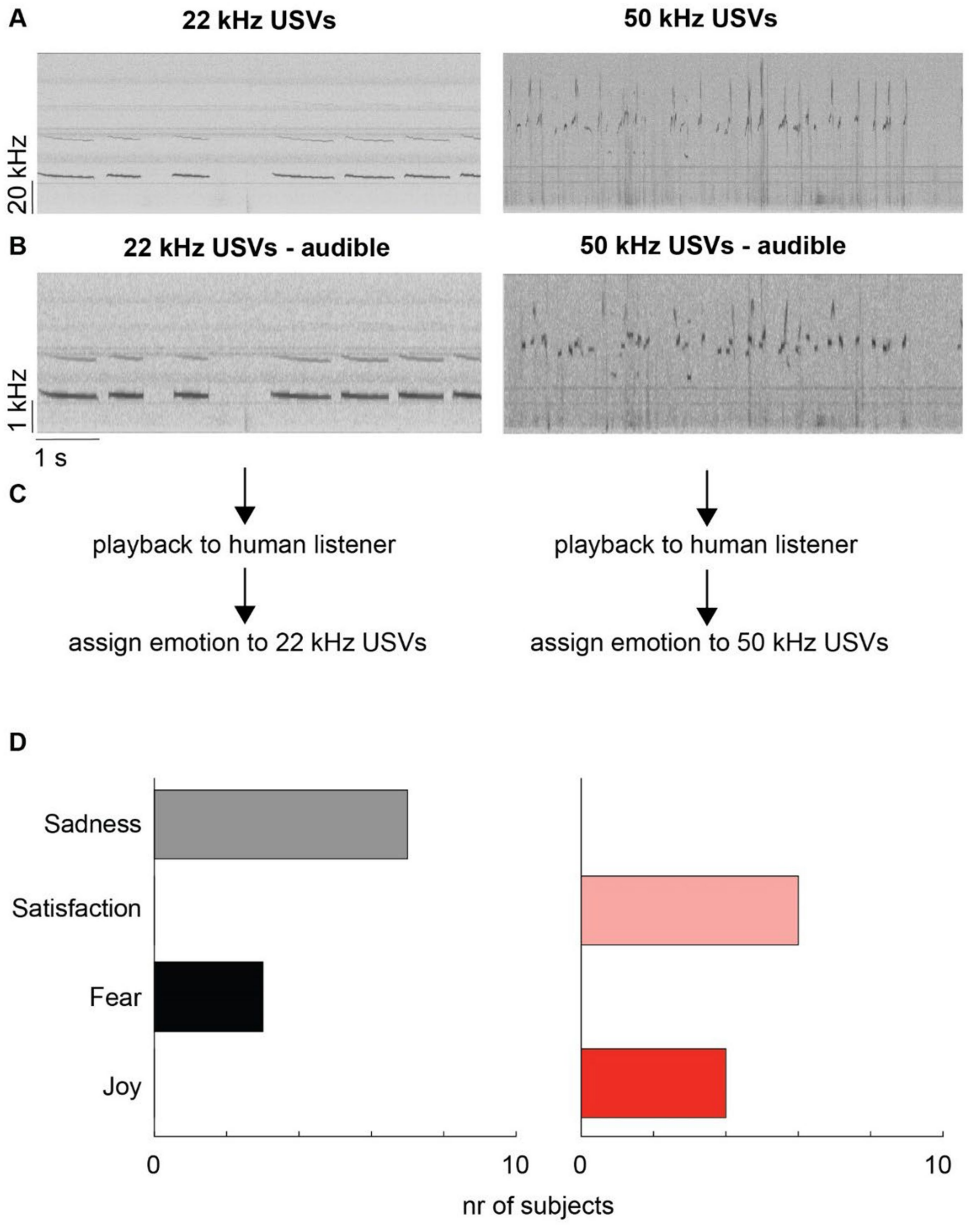
First cohort spectrogram and results. **(A)** For each emotional valence condition one 7 s snipped containing rat USVs was selected. Left: 22 kHz USVs (“fear calls”). Right: 50 kHz USVs (“hedonic calls”). **(B)** Both snippets were transposed to the audible range and were then play-backed to human listeners. **(C,D)** Human listeners (*n* = 10) assigned one out of four emotional states to the perceived playback through an online survey system. Note that 22 kHz USVs were exclusively assigned to negative-valence emotional states (sadness and fear), while 50 kHz USVs were exclusively assigned to positive-valence emotional states (satisfaction and joy).

Two experiments (cohort 1, exploratory/hypothesis generating study and cohort 2, hypothesis testing study) were conducted in succession. USV snippets had different sources for cohort 1 and cohort 2. For cohort 1, they were selected from a published dataset in which one 6-week- old male rat emitted both 22 kHz, as well as 50 kHz USVs following prefrontal cortex activation (Bennett et al., 2019). Note that although these USVs were produced by artificial stimulation they had similar spectro-temporal characteristics as naturally occurring USVs [see (Bennett et al., 2019)]. USVs were frequency shifted similarly as described in Lingle and Riede (2014) and (Schwartz et al., 2024). Specifically, the snippets (each 7 s for cohort 1) were then transposed using Audacity software using the “change speed” function (95% speed reduction). In doing so, vocalization frequencies dropped to the audible range, however, due to the speed reduction they got elongated as well. To retain the temporal structure these elongated snippets were contracted using the “change tempo” function (change length back to 7 s) in the “effects” tab of Audacity. This did not affect frequencies, leading to the ready-for-playback audible snippets shown in Figure 1A. The 22 kHz and 50 kHz snippets contained 7 and 30 vocalizations, respectively. We did not use Audacity’s “change pitch” function, since this method generated unwanted harmonic-like artefacts.

For cohort 2, two new 8.3 s snippets were chosen. 50 kHz USVs were selected from a recording of a 6-week-old male rat tickling session generated in our lab. Tickling consisted of playful interactions between the experimenter’s hand and the rat as described in Ishiyama and Brecht (2016). 22 kHz USVs for cohort 2 were downloaded from the online publication (Granata et al., 2024), in which the authors report that these rat USVs were recorded upon presentation of cat urine. For cohort 2, frequency-shifts were performed as for cohort 1, but to examine whether the degree of frequency transposition affects human perception of emotional valence, we generated three levels of shift: low (97% slowdown), mid (95% slowdown), and high (85% slowdown). All slowed downs were again followed by tempo reduction, such that the snipped retained its original length (8.3 s long).

**Figure 2:**
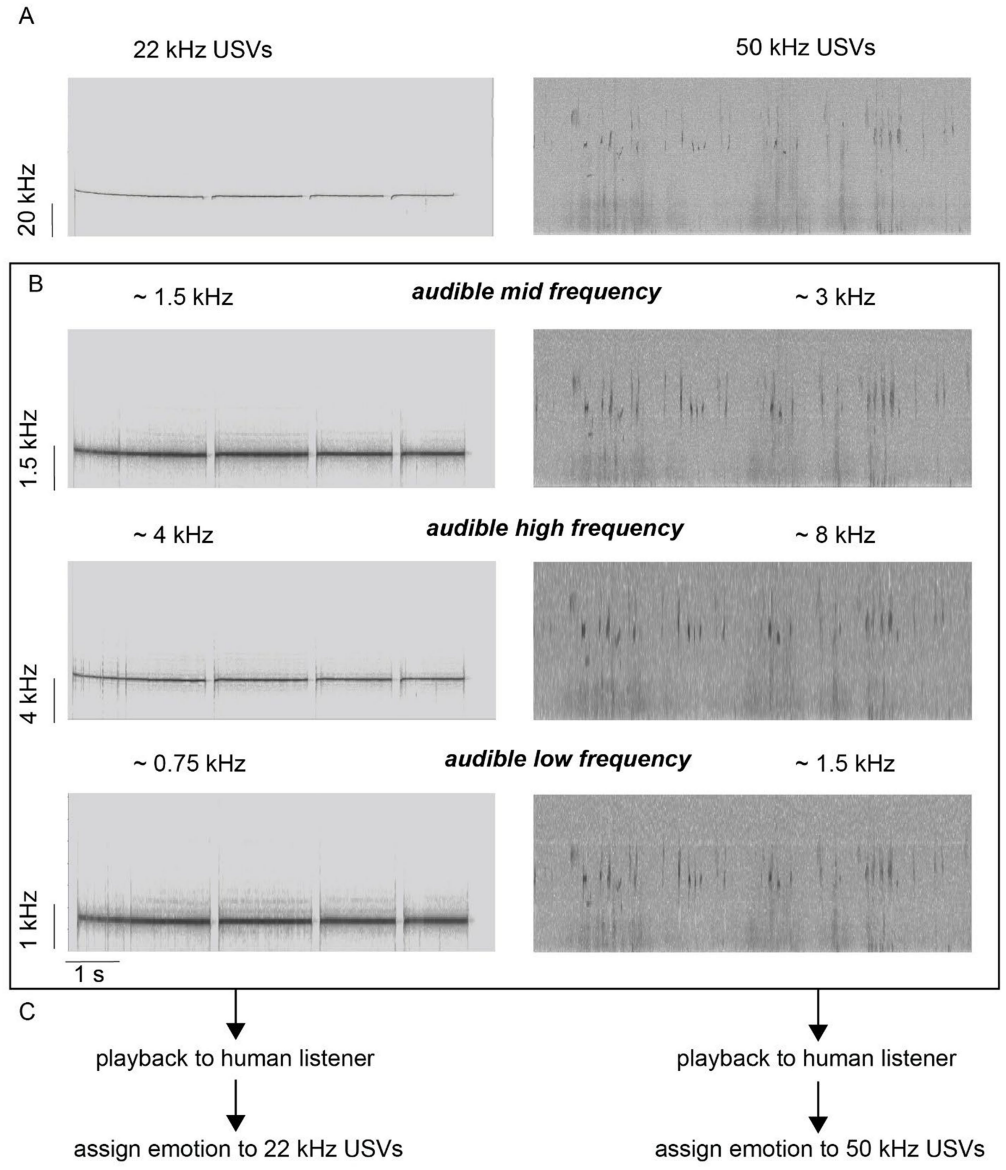
Second cohort spectrograms. **(A)** For each emotional valence condition one ~8 s snipped containing rat USVs was selected. Left: 22 kHz USVs (“fear calls”) emitted following presentation of cat urine (Granata et al., 2024). Right: 50 kHz USVs (“hedonic calls”) emitted during tickling. **(B)** Both snippets were transposed to the audible range and were then play-backed to human listeners. Three variants for each call-type were created with different audible frequencies. For 22-kHz USVs frequencies ranged from ~0.75 kHz to ~4 kHz and for 50 kHz USVs frequencies ranged from 1.5 kHz to 8 kHz. Note that first the mid frequencies were played back, followed by high frequencies and finally by low frequencies. Frequency-shifted vocalizations were played back to participants in the order (from top to bottom) shown in **(C)**.

The different slowdowns resulted in different target (peak) frequencies. For 22 kHz and 50 kHz USVs these were ~0.75 kHz, ~1 kHz, ~4 kHz and ~1.5 kHz, ~3 kHz, ~8 kHz, respectively (see spectrograms in Figure 2 and Supplementary Table 3). We derived peak frequencies by using the “Plot spectrum” function in the “Analyze” tab of Audacity following selection of the whole snippet. The peak of the resulting power spectrum was determined, and the corresponding frequency was noted to obtain the peak frequency values. Note our frequency-shift method retained the ratio between 22 kHz and 50 kHz USVs. We chose these target frequencies as they cover most of the acoustic energy found in human vocalizations (Baeck and de Souza, 2007; Fuller and Horii, 1988; Robb and Cacace, 1995; Zeskind and Lester, 1978) and because in this way some frequency-shifted 22 kHz vocalizations lie below or equal to- and some lie above frequency shifted 50 kHz vocalizations. This allows assessment about to which extend frequency alone may matter or whether other parameters (temporal or amplitude) may matter as well. The 22 kHz and 50 kHz snippets contained 4 and 30 USVs, respectively.

Audible snippets were uploaded to an online survey platform [SoSci Survey (Leiner, 2024)]. The snippets were linked to a short questionnaire with a request to listen to the snippets and subsequently assign the perceived sounds to one of the four emotions: Fear, sadness, joy and satisfaction. In cohort 2 sadness was replaced by concern. For both cohorts subjects above 18 were recruited through social media channels (WhatsApp, Facebook, Email). They were asked to fill out the survey without being given any additional information on the play-backed sounds and the project. Subjects declared consent that their anonymized data will be published. They were also asked to state their professional background or current state of education, as well as their gender, native language and age. Participants were allowed to listen to all playbacks as needed on a given questionnaire page. No spectrograms were shown.

Statistics: Cohort 1 served as an exploratory, hypothesis-generating pilot study. We thus refrained from any statistical analysis. The obtained results suggested that human participants correctly assign emotional valence to rat vocalizations. Thus, in cohort 2 we tested this hypothesis using a Bayesian binomial generalized linear mixed-effects model. A first intercept only model tested whether the overall probability for a correct response exceeded chance by setting subject as random intercept. A second model tested whether correct response rates depended on call type (22 kHz vs. 50 kHz) or on the degree of the frequency shift (‘mid’, ‘high’, ‘low’) by setting these variables as fixed effects and subject as random intercept. A third intercept-only model again with subject as random intercept tested whether participants displayed a bias toward either high or low arousal categories (fear/joy vs. concern/satisfaction, respectively). A link to the code for these statistical analyses is provided in the Supplementary Files section.

## Results

In an initial, exploratory study (cohort 1), we selected two short snippets of rat USVs, one containing negative valence USVs with a few long and downward ramp smooth contours in the 22 kHz range (Figure 1A, left), and the other containing positive valence USVs with many short, frequency-modulated contours in 50 kHz range (Figure 1A, right). We then transposed both snippets into the human audible range, allowing participants to perceive these vocalizations upon playback. While this manipulation altered the absolute frequencies, it preserved the relative spectro-temporal structures of the original USVs (Figure 1B), similarly as shown previously with non-USV vocalizations (Lingle and Riede, 2014). Using an online survey platform, we recruited 10 human participants (cohort 1) without prior exposure to rat vocalizations. Participants were asked to listen to both transposed snippets and assign each sound to one of four emotions: ‘fear’ (high-intensity negative valence); ‘sadness’ (low-intensity negative valence); ‘joy’ (high-intensity positive valence); ‘satisfaction’ (low-intensity positive valence; Figure 1C). Strikingly, as displayed in Figure 1D, all participants correctly assigned the transposed 22 kHz USVs to negative emotions, while 50 kHz USVs were correctly assigned to positive emotions. We refrained from statistics due to the exploratory/hypothesis generating character of this initial study.

To check whether these findings remain qualitatively similar if other USVs from a different context were played-back, and to confirm our initial findings, we conducted a second experiment with new participants (cohort 2, *n* = 19). We also changed the token ‘Sadness’ to ‘Concern’ in order to achieve a balanced set of four emotions differing in both valence and arousal (Russell, 1980; Scherer, 2005). Specifically, ‘concern’ and ‘fear’ share negative valence but differ in intensity level, and the same holds for ‘joy’ and ‘satisfaction’ on the positive side. Finally, we included different frequency shifts (audible mid-frequency, low frequency and high frequency) for play-back (Figure 2). Audible mid-frequency corresponded to the frequency shift performed for cohort 1.

**Figure 3:**
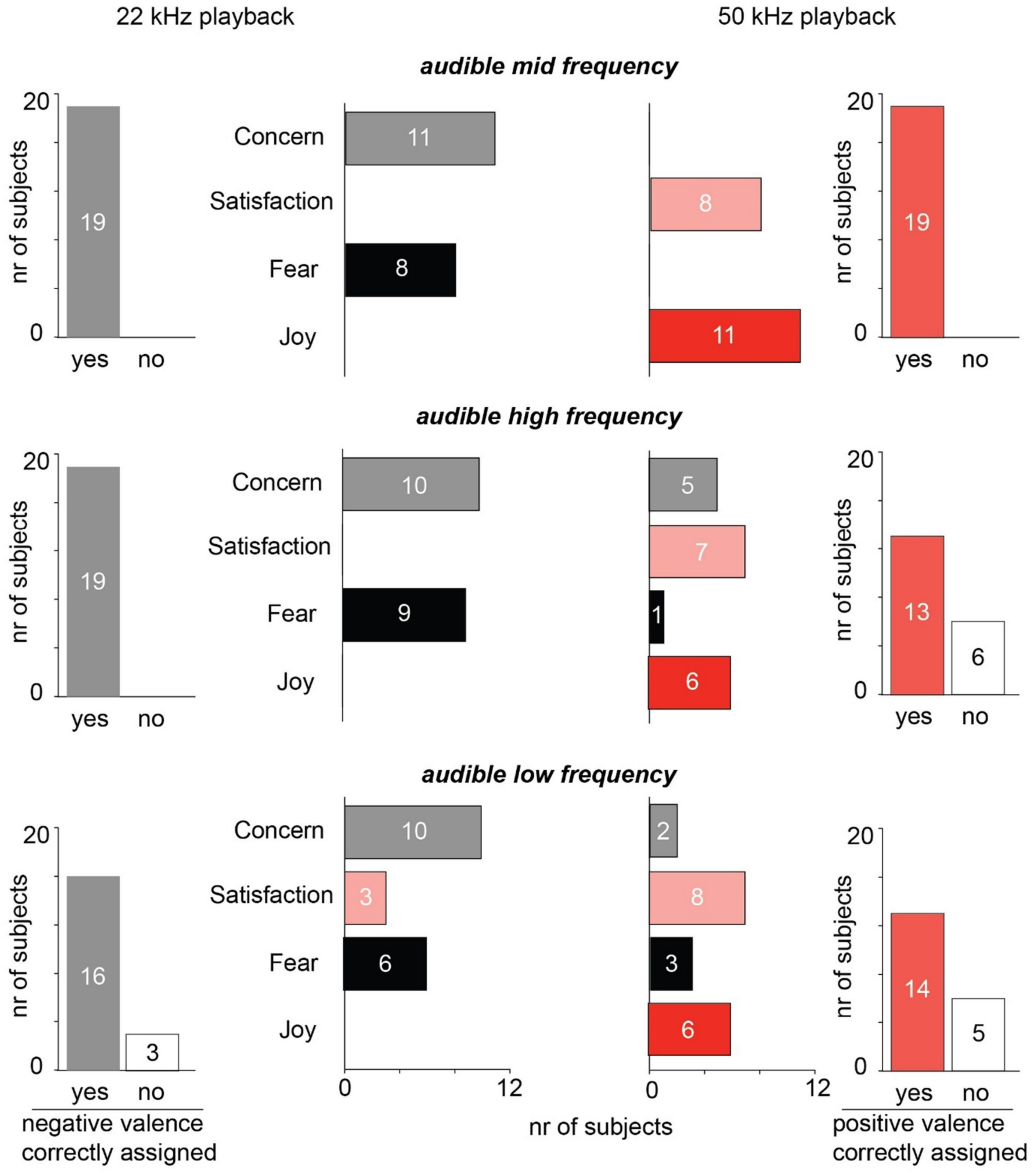
Second cohort results. Human listeners (*n* = 19, different from those of cohort 1) assigned one out of four emotional states to the perceived playback through an online survey system. In this cohort, however, “Sadness” was replaced by “Concern.” Nevertheless, participants again correctly assigned mid frequencies of 22 kHz and 50 kHz USVs to the emotional valence (top row). This effect was less pronounced for the higher and lower played-back frequencies (see Results section for statistical analysis).

For audible mid-frequency playbacks, again, all participants correctly assigned the emotional valence of rat vocalizations for both 22 kHz and 50 kHz playbacks (Figure 3, top). Also, in subsequent negative valence vocalization playbacks with shifts to slightly higher or lower frequencies (‘low’ and ‘high’) most participants correctly assigned rat vocalization valence, but with a smaller correct response rate (Figure 3, left column). A similar effect was observed for the 50 kHZ ‘low’ and ‘high’ condition but with a lower correct response rate compared to the corresponding 22 kHz playbacks (Figure 3, right column).

To test whether correct response rates of this dataset were overall above chance and whether they were statistically different with respect to call type or frequency shift, we fitted the data to a binomial generalized linear mixed effects model. Because the ‘mid’ condition was marked by 100% correct response rates, an initial frequentist binomial generalized linear mixed-effects model resulted in infinite and therefore uninterpretable likelihood estimates due to complete separation. We therefore fitted a Bayesian binomial generalized linear mixed-effects model, which provides finite estimates despite separation (Andrew et al., 2008). A first intercept-only model (Supplementary Table 4) with subject included as a random intercept estimated that the overall probability of a correct response is 0.89. This corresponds to an odds ratio of 7.8, indicating that the odds of an overall correct response were 7.8 times higher than those of an incorrect response. The 95% credible interval (4.5–13.5) did not include 1, demonstrating that participants clearly responded correctly above chance. A second model (Supplementary Table 5) tested whether either call type (22 kHz vs. 50 kHz) or frequency shift (‘low’, ‘mid’, ‘high’) had an influence on participant ratings. Again, subject was set as a random intercept and call type and frequency shift were set as fixed effects. The odds of a correct response were clearly lower for 50 kHz playbacks (odds ratio: 0.32, 95% credible interval: 0.16–0.64). Similarly, correct response rates were lower in the ‘high’ and ‘low’ frequency shift conditions when compared against ‘mid’ frequency shifts (odds ratio: 0.26 and 0.17, respectively; 95% credible intervals: 0.10–0.67 and 0.08–0.40, respectively). Correct response rates were not different from chance when comparing ‘high’ vs. ‘low’ frequency shifts (odds ratio: 1.47, 95% credible interval: 0.59–3.66).

We also tested whether participant ratings showed a bias toward either high or low arousal categories, that is, fear/joy vs. concern/satisfaction, respectively. In our initial pilot study, participants tended to select the lower-arousal categories more frequently (Figure 1). However, since 22 kHz or 50 kHz USVs are often emitted during high arousal contexts (fearful encounters or play), one could expect the opposite, that is, participants rating playbacks as high arousal. To test this hypothesis using the larger dataset obtained from cohort 2, we fitted a third model (intercept-only; Supplementary Table 6), again selecting subject as random intercept. This time, however, we assigned correct responses to high-arousal ratings (fear and joy). The estimated probability of a correct response was 0.43, which was not different from chance (odds ratio: 0.77; 95% credible interval: 0.52–1.12), suggesting that humans did not show any systematic bias toward a certain arousal quality.

Summarized, these results suggest that humans correctly assign emotional valence to rat vocalizations, however, with lower correct response rates for 50 kHz playbacks and frequency shifts that deviated from the ‘mid’ condition.

## Discussion

Several studies showed that humans can correctly assign negative and positive emotional valence to animal vocalizations (Greenall et al., 2022; Scheumann et al., 2017; Tallet et al., 2010; Debracque et al., 2023). Our results extend this understanding by showing that humans can accurately assign emotional valence to rat USVs transposed into the audible range, despite no evolutionary exposure to these frequencies. Rats and humans last shared a common ancestor between 60 and 100 million years ago (Kumar and Hedges, 1998), and while rats have undergone a form of self-domestication prior to laboratory domestication (Munshi-South et al., 2024), they remain evolutionarily distant from humans. Moreover, laboratory rats retain the USV types and spectro-temporal features of wild rats (Kaltwasser, 1990), suggesting that domestication has not altered their vocalizations in a way that would facilitate cross-species emotional recognition. Further, the lack of human ability to perceive rat USVs due to frequency limitations suggests that continuous sensory exposure to these vocalizations is not necessary for their recognition of emotional valence. This finding suggests that vocal emotional recognition is an evolutionarily conserved feature that transcends both phylogenetic distance and natural auditory limitations, which is in line with previous reports showing cross-species emotional content recognition even in species that are evolutionarily more distant than humans and rats (Lingle and Riede, 2014; Filippi et al., 2017). However, it is important to note that in some instances humans confuse the emotional content of rather distant species’ vocalizations (Scheumann et al., 2014).

We also note that in principle prolonged exposure to human vocalizations may have influenced rat vocal repertoires, since human vocalizations are in the hearing range of rats. For this to happen, however, rats must have had at least a gross ability to link human vocal cues to emotional content by model learning or other evolutionary/plasticity mechanisms. Further, such adaptation would require to be evolutionary advantageous, for example, human emotional vocalizations would needed to be superior in reliably signaling internal or environmental conditions. While speculative, this scenario cannot be entirely excluded.

Further, theories on emotional sound perception suggest that certain rules may be generalized across species (Ehret, 2006; Korcsok et al., 2024; Morton, 1977). This raises the hypothesis that the valence of human vocalizations, such as crying and laughter, may be correctly interpreted by rats, which could be tested in an analogous but reverse experiment. Emotional cues may be encoded in universal acoustic features independent of a species’ frequency range. For example, musical intervals associated with sadness (minor second and -third) have been linked to correspondingly sad emotions (Curtis and Bharucha, 2010; Zeloni and Pavani, 2022), indicating that the (logarithmic-) frequency ratios may carry significant information about emotional content.

Nevertheless, absolute frequencies may be relevant within a species’ hearing range. Therefore, we chose frequencies of our transposed USVs in a way that would correspond to a broad range of the human vocalizations. For example, infant cries show acoustic energy between 0.3 and 5 kHz (Baeck and de Souza, 2007; Fuller and Horii, 1988; Robb and Cacace, 1995; Zeskind and Lester, 1978). Also, adult emotional vocalizations carry significant spectral energy in this range (Sauter et al., 2010; Arnal et al., 2015; Bachorowski and Owren, 2001). High- and low frequency play-backs of positive-valence rat vocalizations seemed to be more ambiguous to human participants, which aligns with studies suggesting that vocalizations associated with negative affect are stronger preserved through evolution (Kamiloğlu et al., 2023; Kamiloğlu et al., 2020). We made sure that at least two target frequencies for 50 kHz playback [~1.5 kHz (“low”) and ~3 kHz (“mid”)] are lower than the highest 22 kHz playback frequency (~4 kHz). Both high-frequency and low-frequency playbacks resulted in lower rates of correct participant ratings. Together these results suggest that the absolute playback frequency may influence correct emotional assessment of perceived vocalizations. However, as other acoustic features, such as duration and amplitude have been shown to be important predictors for valence categorization (Lefèvre et al., 2025; Maigrot et al., 2018; Friel et al., 2019; Korcsok et al., 2020; Yin and McCowan, 2004; Taylor et al., 2009) future experiments should perform similar playbacks with manipulations of aforementioned features. Finally, as impaired ratings only were observed after the initial ‘mid frequency’ playback, we cannot entirely rule out that ratings may be dependent on the sequence in the questionnaire. Thus, further experiments should be conducted in which both spectral- and temporal features, as well as their sound pressure levels are manipulated and played-back in a randomized fashion.

Regarding perceived arousal, our initial, exploratory pilot study (cohort 1) showed a tendency toward ratings of low arousal categories (fear/joy vs. concern/satisfaction), although clearly less absolute than for the valence attributions. We were surprised by this initial observation since rats emit 22 kHz and 50 kHz in rather high arousal contexts (during fearful encounters or during play – see Introduction). We therefore re-examined this issue with the larger dataset obtained from cohort 2. Our statistical test did not reveal a systematic bias of ratings toward high or low arousal categories, suggesting that, unlike emotional valence, arousal may not be as readily decoded by humans.

A limitation of our study is the relatively low number of participants and the low number of playback snippets. Although the effect regarding the overall claim of our study was striking and statistically sound, further playback studies should present a variety of playbacks to increase generalizability.

Another limitation of our study is that participants were allowed to listen to all playbacks on a given questionnaire page, which may have encouraged comparison between playbacks. Future studies should present playbacks individually to minimize potential comparison effects.

Previous studies noted similarities between rat vocalizations and human emotional expressions, supported by resemblances in spectro-temporal features between human laughter and 50 kHz USVs, as well as human crying and 22 kHz USVs (Panksepp and Burgdorf, 2003; Brudzynski, 2019). Our study provides empirical data showing that humans are able to correctly assign putative emotional categories of rat vocalizations, even when these originate outside the natural hearing range.

## Data Availability

Data will be shared upon request via email (eduard.maier@zi-mannheim.de). Vocalization snippets can be accessed on figshare through the following links: Cohort 1. 22 kHz: https://figshare.com/s/64f36ab72c2fa06567e2. 22 kHz – audible: https://figshare.com/s/18aa657a5a9a67081e43. 50 kHz: https://figshare.com/s/cadcc4e5f9a3be6872b5. 50 kHz – audible: https://figshare.com/s/73172a1637c318b070ab. Cohort 2. All files: https://doi.org/10.6084/m9.figshare.30177595.v1.
