# Supplementary figures and images for: Humans correctly assign emotional valence of rat vocalizations
Source: Front Psychol. 2026 May 20;17:1769385. doi: 10.3389/fpsyg.2026.1769385 (PMC13230224; doi:10.3389/fpsyg.2026.1769385)

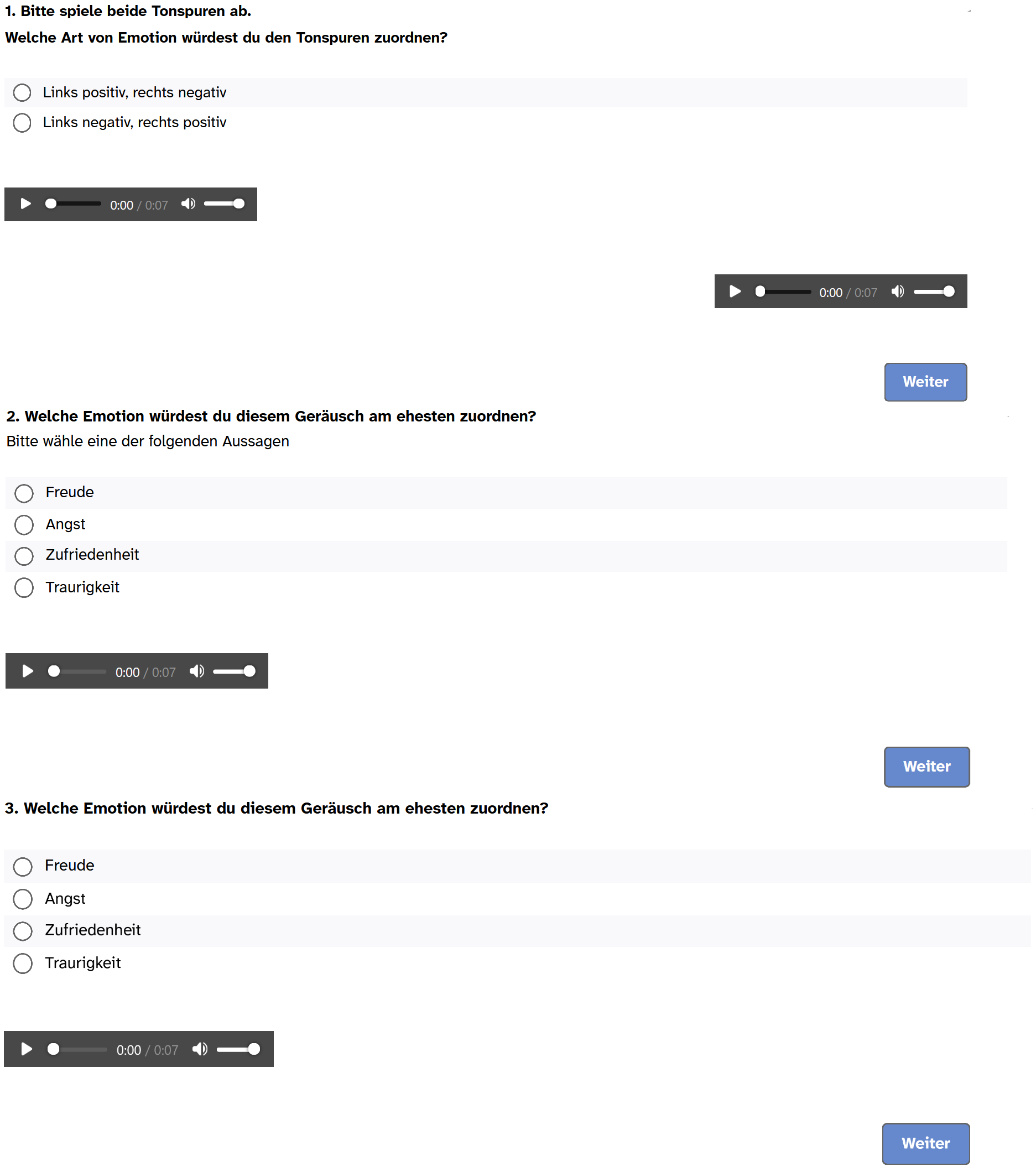

Supplement: Supplementary file 1 [file Image_1.png]

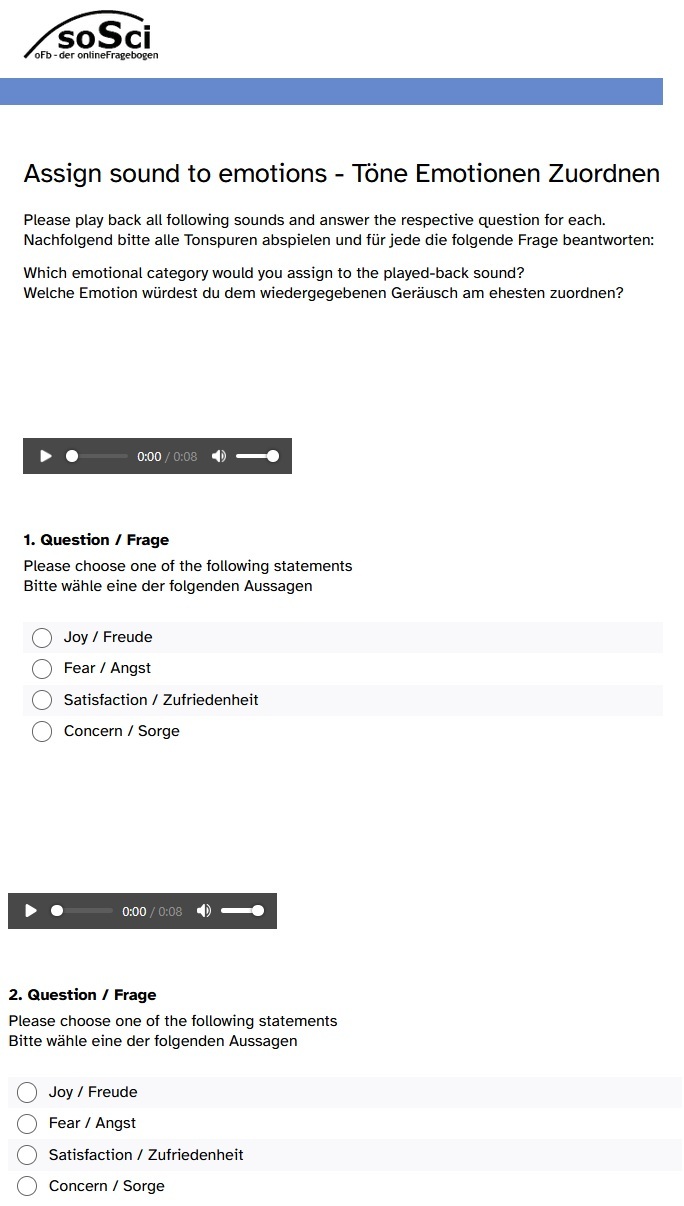

Supplement: Supplementary file 2 [file Image_2.jpg]

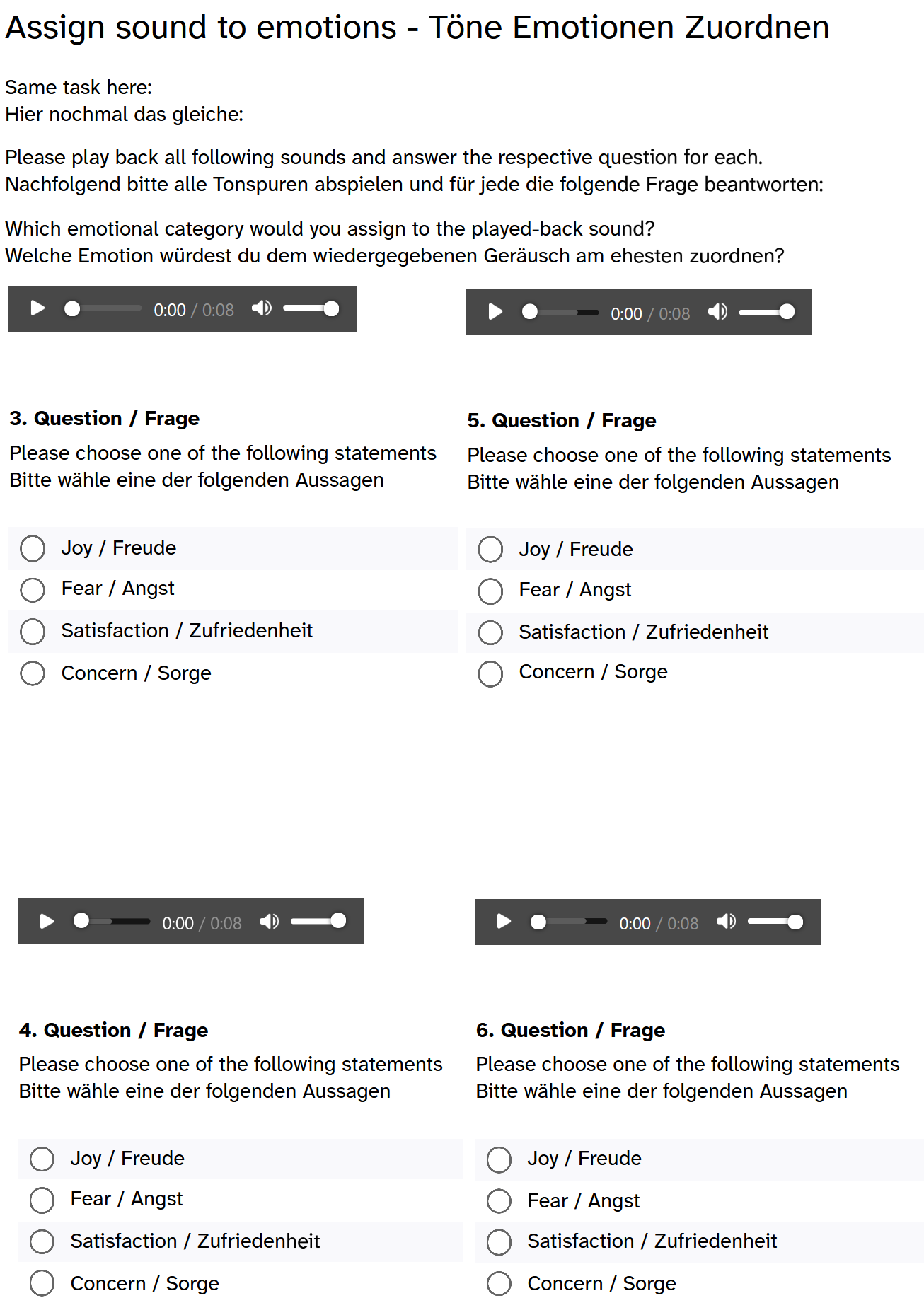

Supplement: Supplementary file 3 [file Image_3.png]
