# Supplementary material for: Humans correctly assign emotional valence of rat vocalizations
Source: Front Psychol. 2026 May 20;17:1769385. doi: 10.3389/fpsyg.2026.1769385 (PMC13230224; doi:10.3389/fpsyg.2026.1769385)
Supplement: Supplementary file 4 [file Supplementary_file_1.docx]

**Supplementary Files.**

Cohort 1

22 kHz: <https://figshare.com/s/64f36ab72c2fa06567e2>

22 kHz - audible: <https://figshare.com/s/18aa657a5a9a67081e43>

50 kHz: <https://figshare.com/s/cadcc4e5f9a3be6872b5>

50 kHz - audible: <https://figshare.com/s/73172a1637c318b070ab>

Cohort 2

All files: <https://doi.org/10.6084/m9.figshare.30177595.v1>

Filenames

22 kHz: 22kHZ_snipped_granataEtAl.wav

22 kHz - audible-mid 22kHZ_snipped_granataEtAl_audible95prcntRdct.mp3

22 kHz - audible-low 22kHZ_snipped_granata_audible97prcntRdct-2.mp3

22 kHz - audible-high 22kHZ_snipped_granataEtAl_audible85prcntRdct.mp3

50 kHz: 50kHz_snippet_6min15to6min25s.wav

50 kHz - audible-mid 50kHz_snippet_6min15to6min25s_95prctRdct_8.27s.mp3

50 kHz - audible-low 50kHz_snippet_6min15to6min25s_97prctRdc_8.27s.mp3

50 kHz - audible-high 50kHz_snippet_6min15to6min25s_85prctRdct_8.27s.mp3

**Code file for Bayesian binomial generalized linear mixed-effects model**

<https://figshare.com/s/466f7c81d1ec89f7085c>

(10.6084/m9.figshare.31376008)

**Data table ued for Bayesian binomial generalized linear mixed-effects model**

<https://figshare.com/s/d4630f31c18e6cf33a28>

10.6084/m9.figshare.31533421

**Supplementary Table 1 (Cohort 1)**

| **Age range (years)** | **Gender** | **Native language** | **Professional background / education** |
| --- | --- | --- | --- |
| 45 - 49 | Female | Russian (German fluent) | Nurse |
| 50 - 54 | Male | Russian (German fluent) | Bus driver |
| 20- 24 | Male | German | IT specialist |
| 65 - 69 | Female | Russian (German fluent) | House wife |
| 30 - 34 | Male | German | Physician |
| 40 - 44 | Female | Russian (German fluent) | Physician |
| 75 - 79 | Male | Russian (German fluent) | Physician |
| 40 - 44 | Male | German | Physician |
| 30 - 34 | Male | German | Medical student |
| 25 - 30 | Male | German | Mechanic |

Note: Age is given in range for deidentification reasons.

**Supplementary Table 2 (Cohort 2)**

| **Age (years)** | **Gender** | **Native language** | **Professional background / education** |
| --- | --- | --- | --- |
| 35 - 39 | Female | German | Key Account Manager |
| 35 - 39 | Male | Italian | Financial Analyst |
| 35 - 39 | Female | German | Social Worker |
| 25 - 29 | Non-specified | Croatian | PhD Student Chemistry |
| 25 - 29 | Female | Italian | PhD Student |
| 20 - 24 | Female | Portuguese | Undergraduate Student Physiotherapy |
| 40 - 44 | Male | German | Bank employee |
| 70 - 74 | Male | German | Pensioner |
| 65 - 69 | Female | German | Pensioner |
| 35 - 39 | Male | German | Management Consulting |
| 25 - 29 | Female | German | Scientific Assistant |
| 40 - 44 | Male | German | IT Consultant |
| 65 - 69 | Male | German | Pensioner |
| 60 - 64 | Female | German | House Wife |
| 30 - 34 | Male | Swedish | Logistics Planner |
| 30 - 34 | Male | Romanian / Italian | Pricing Manager |
| 25 - 29 | Female | Hungarian | Principal Analyst (Supply Chain Risk Management) |
| 55 - 59 | Female | Hungarian | Project Manager |
| 35 - 39 | Male | Hungarian | Pilot Instructor |

**Supplementary Table 3 - Peak Frequency (kHz)**

|  | Original (USV) | ‘mid’ | ‘high’ | ‘low’ |
| --- | --- | --- | --- | --- |
| 22 kHz cohort 1 | 23.4 | 1.2 | - | - |
| 50 kHz cohort 1 | 55.5 | 3.0 | - | - |
| 22 kHz cohort 2 | 22.9 | 1.1 | 3.4 | 0.7 |
| 50 kHz cohort 2 | 54.6 | 2.7 | 8.2 | 1.6 |

**Supplementary Table 4 – Model 1 (testing valence)**

|  | **Type** | **Post. Mean** | **Post. SD** | **SD** | **SD (LB)** | **SD (UB)** |
| --- | --- | --- | --- | --- | --- | --- |
| **Intercept** | M | 2.0570 | 0.2838 |  |  |  |
| **subject** | V | -0.5822 | 0.1626 | 0.559 | 0.404 | 0.773 |

Parameter types are mean structure (M) and variance structure (V)
Variance parameters are modeled as log standard deviations

**Supplementary Table 5 – Model 2 (testing valence and compare call type and frequency shifts)**

|  | **Type** | **Post. Mean** | **Post. SD** | **SD** | **SD (LB)** | **SD (UB)** |
| --- | --- | --- | --- | --- | --- | --- |
| **Intercept** | M | 3.9828 | 0.2981 |  |  |  |
| **C(recording)[T.50kHz]** | M | -1.1515 | 0.3620 |  |  |  |
| **C(frequency, Treatment(reference="mid"))[T.high]** | M | -1.3324 | 0.4737 |  |  |  |
| **C(frequency, Treatment(reference="mid"))[T.low]** | M | -1.7489 | 0.4277 |  |  |  |
| **Subject** | V | -0.4484 | 0.1620 | 0.639 | 0.462 | 0.883 |

Parameter types are mean structure (M) and variance structure (V)
Variance parameters are modeled as log standard deviations

**Supplementary Table 6 – Model 3 (testing arousal)**

|  | **Type** | **Post. Mean** | **Post. SD** | **SD** | **SD (LB)** | **SD (UB)** |
| --- | --- | --- | --- | --- | --- | --- |
| **Intercept** | M | -0.2653 | 0.1951 |  |  |  |
| **subject** | V | -0.4605 | 0.1621 | 0.631 | 0.456 | 0.873 |

Parameter types are mean structure (M) and variance structure (V)
Variance parameters are modeled as log standard deviations
